# Quantitative Analysis of the Vitamin D_3_ Content in Dietary Supplements Marketed in Hungary Using High-Performance Liquid Chromatography

**DOI:** 10.3390/ph19030493

**Published:** 2026-03-17

**Authors:** András Nagy, Róbert György Vida, Eszter Fliszár-Nyúl, Gábor Lovász, Katalin Fábián, Gábor Pozsgai

**Affiliations:** 1 Department of Pharmacology, Faculty of Pharmacy, University of Pécs, 7624 Pécs, Hungary; nyul.eszter@gytk.pte.hu (E.F.-N.); lovasz.gabor@edu.pte.hu (G.L.); fabian.kata@gytk.pte.hu (K.F.); pozsgai.gabor@gytk.pte.hu (G.P.); 2 Department of Pharmaceutics, Faculty of Pharmacy, University of Pécs, 7624 Pécs, Hungary; vida.robert@pte.hu

**Keywords:** vitamin D_3_, HPLC, dietary supplements, product labeling, consumer product safety, quality, patient education, photostability

## Abstract

**Background/Objectives:** The use of over-the-counter vitamin D_3_ supplements has increased substantially in recent years. Compared with pharmaceuticals, dietary supplements are subject to less stringent regulatory oversight, raising concerns regarding labeling accuracy, consumer knowledge, and patient safety. This study aimed to assess public knowledge and preferences related to vitamin D_3_ supplementation and to evaluate the content accuracy and short-term stability of commonly used products. **Methods:** A cross-sectional online survey containing 39 questions was conducted in Hungary between 1 May and 30 June 2024. Based on survey responses, the most frequently used vitamin D_3_ supplements (five soft gel capsules and four tablets) were selected for laboratory analysis. Vitamin D_3_ content was quantified using a validated high-performance liquid chromatography (HPLC) method with UV detection. Soft gel capsules were additionally exposed to natural daylight for one month to assess short-term photostability. **Results:** In total, 367 participants (mean age 31.0 ± 12.5 years) completed the survey, and only 3.5% answered correctly all knowledge-based questions. Six commonly reported supplement brands accounted for approximately 90% of responses. Measured vitamin D_3_ content remained within the tolerance limit (−20% to +50%). Following sunlight exposure, three of four capsule products showed no substantial vitamin D_3_ loss, while one exhibited a 14.7% decrease. **Conclusions:** Most analyzed vitamin D_3_ supplements complied with labeled content claims, but substantial knowledge gaps were identified that may affect patient safety. The validated HPLC method supports pharmacovigilance-oriented quality monitoring of vitamin D_3_ supplements and underscores the need for improved professional counseling.

## 1. Introduction

Vitamin D_3_ is a fat-soluble steroid hormone essential for calcium and phosphate homeostasis and skeletal health [1]. The two major forms relevant to humans are vitamin D_2_ (ergocalciferol), mainly of plant origin, and vitamin D_3_ (cholecalciferol), synthesized in the skin under ultraviolet B (UVB) radiation or obtained from animal-derived foods and supplements. Both forms are hydroxylated in the liver to 25-hydroxyvitamin D [25(OH)D], the primary circulating biomarker of vitamin D status, and subsequently converted in the kidneys to the biologically active 1,25-dihydroxyvitamin D [1,25(OH)_2_D], which binds to the vitamin D receptor (VDR) in multiple tissues [2]. Through this pathway, vitamin D_3_ regulates intestinal calcium absorption, bone remodeling, immune function, and cellular differentiation. Deficiency has been linked to rickets, osteomalacia, and increased susceptibility to autoimmune and infectious diseases [3].

Although its role in bone health is well established, research in recent decades has increasingly focused on potential non-skeletal effects of vitamin D_3_. Observational studies have suggested associations between adequate vitamin D_3_ status and a reduced risk of cardiovascular disease (CVD), possibly through modulation of blood pressure, endothelial function, and inflammatory pathways [4]. Vitamin D_3_ has also been implicated in glucose homeostasis and insulin sensitivity, with deficiencies linked to an increased risk of type 2 diabetes and metabolic syndrome, although trials have produced inconsistent results [5]. Furthermore, the anti-proliferative and pro-differentiation effects of vitamin D_3_ have been the subject of extensive study. These properties have been most consistently demonstrated in immune-mediated inflammatory diseases, such as psoriasis [6]. Vitamin D analogues are used to treat these diseases due to their immunomodulatory effects on T-cell responses and keratinocyte differentiation. In contrast, evidence for similar effects in oncology, particularly with regard to colorectal, breast, and prostate cancers, remains inconsistent, and causal relationships have yet to be firmly established [7].

Vitamin D_3_ deficiency is common in Europe. Serum 25-hydroxyvitamin D < 50 nmol/L (20 ng/mL) is found in less than 20% of the population in Northern Europe, whereas in Western, Southern, and Eastern Europe, the prevalence ranges between 30–60% [8]. Severe deficiency (<30 nmol/L or 12 ng/mL) affects more than 10% of the European population [8]. The problem is particularly pronounced in Hungary, where critically low intake of cholecalciferol has been reported across the general population. Both men and women consume less than half of the nationally recommended daily amount of 5 μg. The situation is especially critical among women over 65 years of age, whose average intake reaches only 38% of the recommended dose. According to the 2009 Hungarian National Nutrition and Nutritional Status Survey, the average daily intake was 2.5 ± 0.07 μg for men and 1.9 ± 0.04 μg for women, with the sex difference being statistically significant. Both men and women showed the highest daily intake in the 18–34 age group, whereas the elderly group (≥65 years) had the lowest vitamin D_3_ intake [9].

Due to this widespread deficiency, the use of dietary supplements has become increasingly relevant. Motivations include prevention of nutrient deficiencies, improvement of overall health, management of specific conditions, and enhancement of performance. Clinical guidelines also note potential benefits in reducing falls and fractures in older adults and modulating immune responses [10]. Supplement use increased during the COVID-19 pandemic, though evidence for prevention of infection is uncertain [11]. As supplement use grows, the potential for drug-supplement interactions rises, which may alter drug efficacy or increase adverse effects [12].

Several analytical techniques have been developed for the determination of vitamin D_3_ in foods, pharmaceuticals, and dietary supplements [13,14,15]. High-performance liquid chromatography with ultraviolet detection (HPLC-UV) has long been used for the routine quantification of vitamin D compounds due to its robustness and suitability for lipid-based matrices [14]. More recently, advanced techniques such as liquid chromatography–mass spectrometry (LC-MS) and LC-MS/MS have also been applied to improve sensitivity and selectivity, particularly for complex biological samples [16]. However, HPLC-UV remains a widely accessible and reliable method for routine quality control of commercial vitamin D_3_ products. In the present study, an HPLC-UV method adapted from previously published procedures was applied to determine vitamin D_3_ content in dietary supplements [14].

Therefore, the aim of this study was twofold: (1) to conduct an analytical evaluation of selected vitamin D_3_ dietary supplements marketed in Hungary, assessing their compliance with declared label values, and (2) to survey consumers in order to gauge knowledge and usage patterns of vitamin D_3_.

## 2. Results

### 2.1. Public Survey

The questionnaire was available for 2 months online between 1 May 2024 and 30 June 2024 and during this period, 367 participants completed our survey, including 251 women and 116 men. 221 participants were younger than 30 years old (mean: 31.36 years old, SD: 12.48). There were participants from every county. Approximately half of respondents (49.9%) had a higher education (college/university degree), while 43.1% had only secondary (high school) education (Table 1).

Five true/false statements assessed knowledge related to dietary supplementation, and only 3.5% of participants answered all items correctly (Table 2). Notably, one statement addressing the role of dietary supplements in disease prevention and treatment was answered incorrectly by approximately 80% of respondents, indicating a widespread misconception.

**Figure 1:**
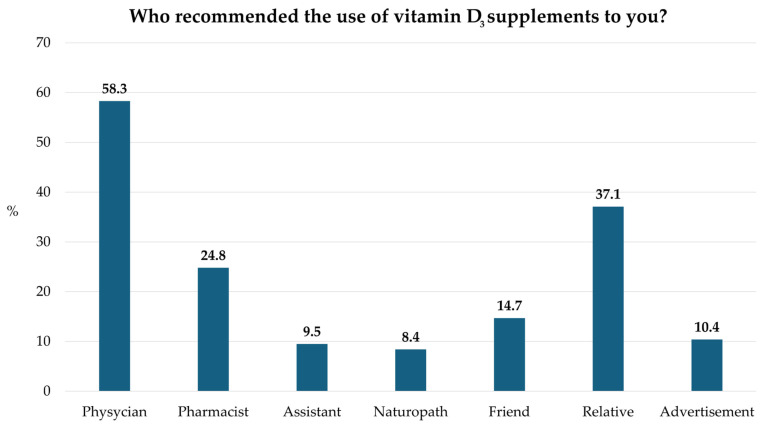
Sources of vitamin D_3_ supplement recommendations (*n* = 367). Bars represent the proportion (%) of respondents who reported each source of recommendation. Multiple responses were permitted.

The third section was about the purchasing habits regarding vitamin D_3_ supplements (Figure 1). When asked who recommended taking vitamin D_3_, the majority cited physicians, followed by family members/relatives, and only 24.8% mentioned pharmacists.

**Figure 2:**
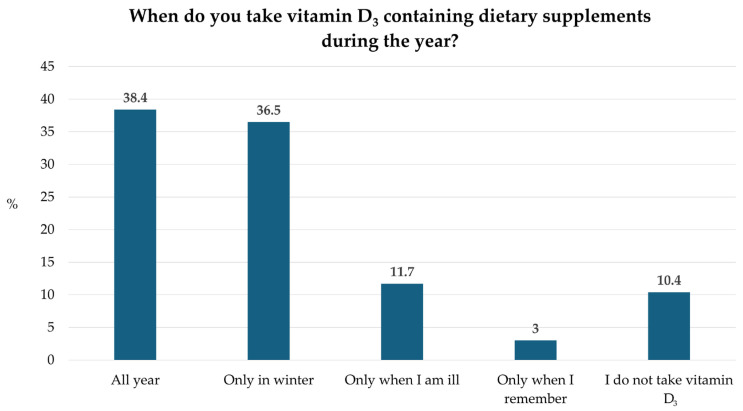
Timing of vitamin D_3_ supplement intake throughout the year (*n* = 367). Bars represent the percentage (%) of respondents reporting each supplementation pattern.

The main factors affecting the selection of a vitamin D_3_ supplement were recommendations from professionals, price and brand. Of the respondents, 38.4% take vitamin D_3_ throughout the year, 36.5% only take it during winter for preventive purposes, and 10.4% do not use vitamin D_3_ at all (Figure 2).

**Figure 3:**
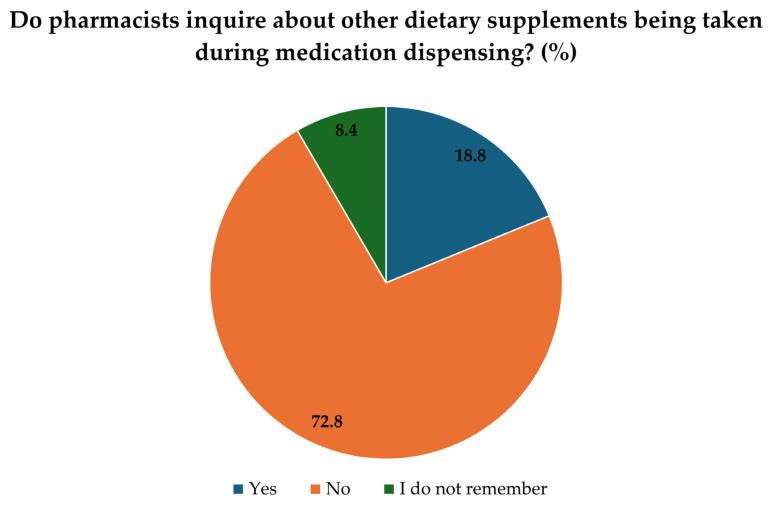
Inquiry of pharmacists about other dietary supplements during dispensing (*n* = 367). Values represent the percentage (%) of respondents who reported whether pharmacists routinely asked about the use of other dietary supplements during medication dispensing.

The final section of the survey focused on questions related to medication use. Of the respondents, 21.8% reported having a chronic illness. Additionally, 12.8% indicated that they take vitamin D_3_ that has been prescribed by a doctor. We investigated whether pharmacists ask about other supplements when dispensing medication, and only 8.4% of respondents said that this had happened during their visit to the pharmacy (Figure 3).

### 2.2. Validation Results

#### 2.2.1. Suitability

**Figure 4:**
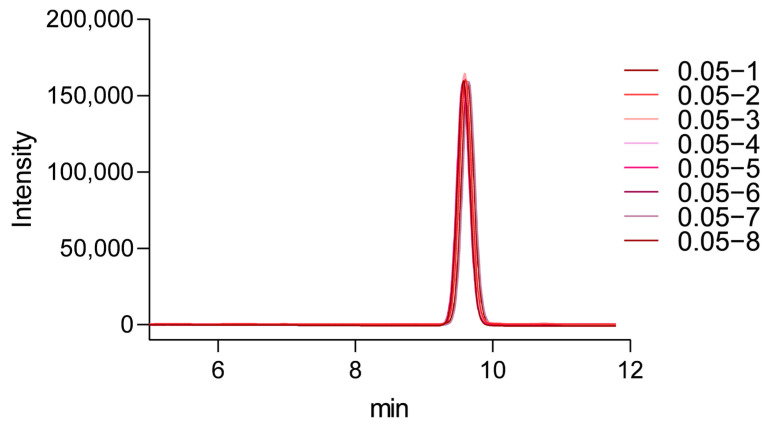
Suitability of 0.05 mg/mL vitamin D_3_ standard solution. Overlaid chromatograms demonstrate consistent retention time and peak shape across replicate injections. The retention time of vitamin D_3_ was 9.6 min.

Eight consecutive injections were performed using a 0.05 mg/mL vitamin D_3_ standard solution (Figure 4). The retention time for vitamin D_3_ was consistently observed at 9.6 min, with a relative standard deviation (RSD) of 0.306%. Peak area reproducibility was acceptable, with an RSD of 1.95% and symmetry factor (As) values remained within the acceptable limit (As < 1.5). These results confirmed the system’s reliability and suitability for subsequent analytical measurements.

#### 2.2.2. Linearity and Range

**Figure 5:**
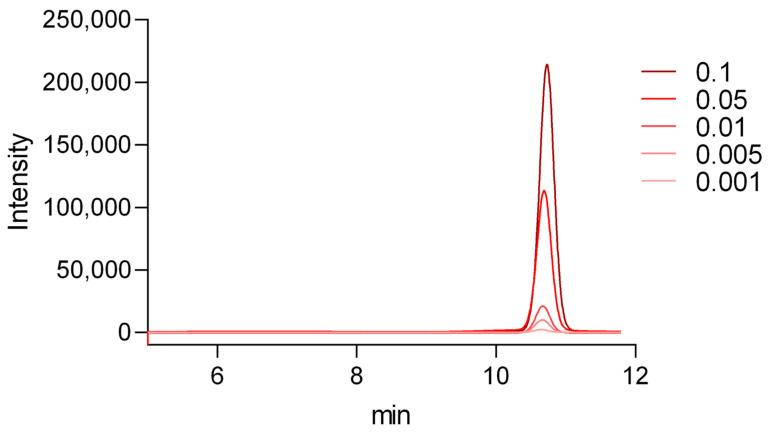
The linearity of the method using standard concentrations from 0.1 mg/mL to 0.001 mg/mL. Representative chromatograms obtained at concentrations between 0.001 and 0.1 mg/mL show a concentration-dependent increase in signal intensity, confirming linear detector response within the validated range.

The linearity of the method was assessed using five standard concentrations: 0.1 mg/mL, 0.05 mg/mL, 0.01 mg/mL, 0.005 mg/mL, and 0.001 mg/mL (Figure 5). A calibration curve was constructed by plotting the peak area against the concentration of vitamin D_3_.

**Figure 6:**
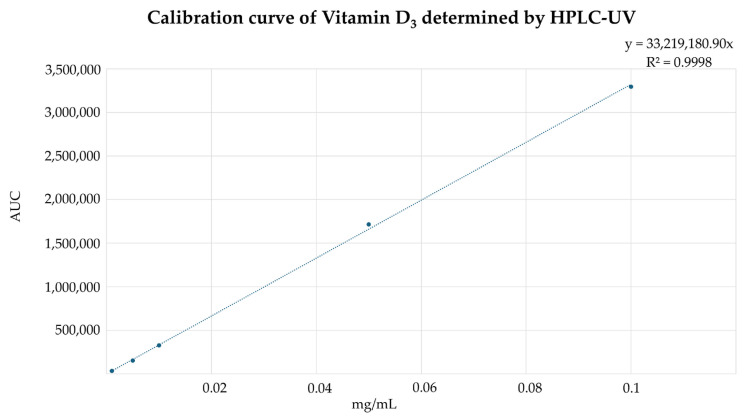
Calibration curve and regression analysis of the HPLC method for vitamin D_3_ quantification. The plot shows the linear relationship between peak area (AUC = area under the curve) and vitamin D_3_ concentration (mg/mL) over the validated range, with a coefficient of determination (R^2^) of 0.9998.

The regression analysis showed an R-value of 0.9998, indicating excellent linearity (Figure 6). This demonstrates that the method reliably quantifies vitamin D_3_ within this range. The range of the method, defined as the interval over which linearity, precision, and accuracy are maintained, was confirmed between 0.001 mg/mL and 0.1 mg/mL.

#### 2.2.3. Repeatability, LOD and LOQ

**Figure 7:**
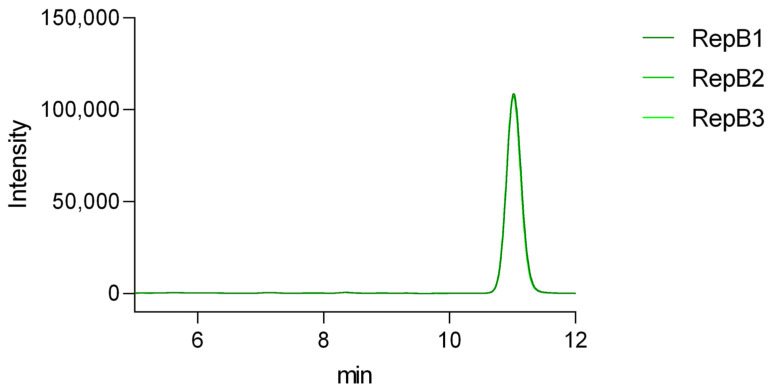
Repeatability of the method using a 0.05 mg/mL vitamin D_3_ standard. Overlaid chromatograms represent three replicate analyses performed on three consecutive days, demonstrating consistent retention time and peak shape, indicative of good intra- and inter-day repeatability.

The repeatability of the method was evaluated using our standard vitamin D_3_ solution at a concentration of 0.05 mg/mL (Figure 7). The measurement was performed three times per day over three consecutive days (mean peak area: 1,857,022; SD: 28,241). Peak area reproducibility was acceptable, with RSD values of 1.31%, 1.22%, and 1.51% for the individual days, and an overall RSD of 1.52%. Symmetry factor (As) values remained within the acceptable limit (As < 1.5). The results showed consistent values across replicates and days, indicating good repeatability of the method (Figure S3).

The method showed a limit of detection (LOD) of 5 × 10^−7^ mg/mL and a limit of quantification (LOQ) of 5 × 10^−6^ mg/mL, indicating high sensitivity and suitability for trace vitamin D_3_ measurements (Figures S1 and S2).

### 2.3. HPLC Measurements

#### 2.3.1. Capsules

**Figure 8:**
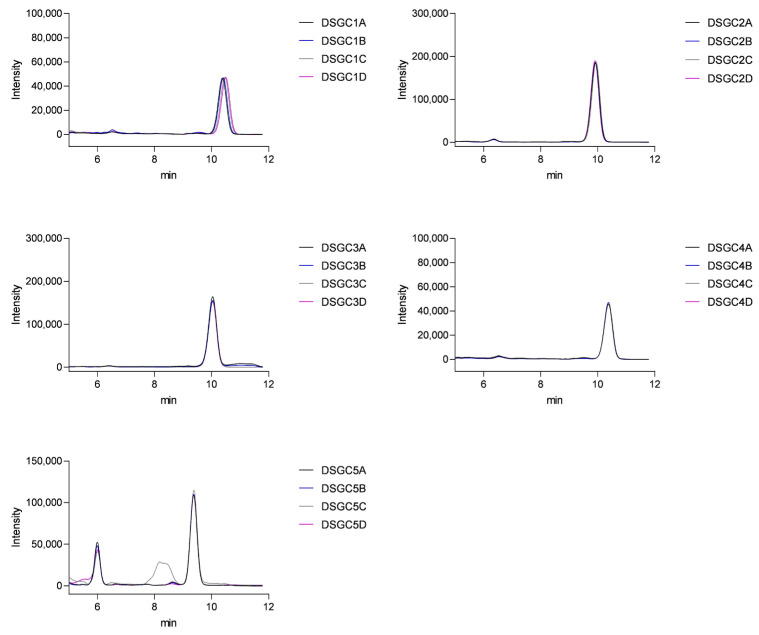
Representative HPLC–UV chromatograms of vitamin D_3_ dietary supplement gel capsule samples (DSGC1–DSGC5). DSGC denotes dietary supplement gel capsule. The main peak corresponds to vitamin D_3_, identified based on retention time and UV absorption spectrum by comparison with a reference standard. Panels A–D show four repeated injections of each preparation to illustrate chromatographic repeatability.

For each preparation, the average was determined from four samples. The chromatograms displayed a distinct peak for vitamin D_3_ with a retention time of approximately 10 min (Figure 8). The absence of significant interfering peaks indicates good specificity of the method.

The obtained results were compared with the values indicated on the packaging. The measured concentrations of vitamin D_3_ in the supplements ranged from 50 to 75 µg per serving. According to the EU guidance on tolerances for nutrient values declared on a label, vitamins in food supplements may be tolerated within −20% to +50% of the declared value [18]. In the context of this study, all analyzed products remained within these regulatory tolerance ranges (Table 3).

##### Stability of Capsules After One Month of Daylight Exposure

After one month of daylight exposure, re-analysis of the samples showed that three of the four tested supplements retained vitamin D_3_ content similar to the initial measurements. However, in the case of DSGC2, a significant decrease was observed: the measured content declined from 89.45% at baseline to 76.32% after one month, which represents a 14.7% relative loss of active ingredient (Table 4). Further experimental details are provided in the materials and methods section.

#### 2.3.2. Tablets

**Figure 9:**
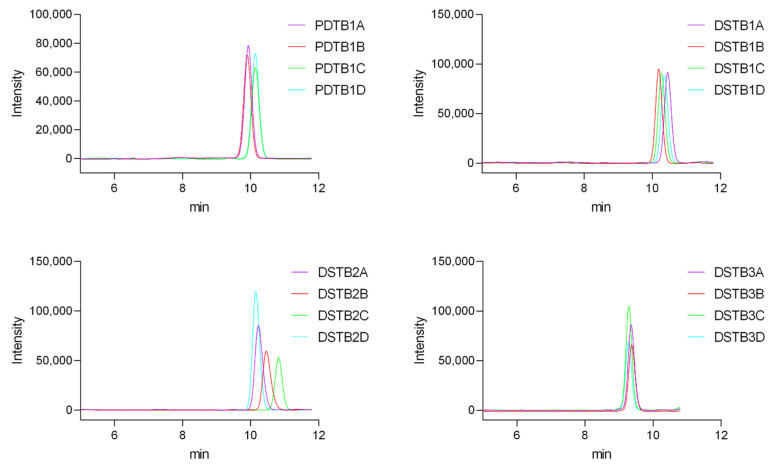
Representative HPLC–UV chromatograms of tablet formulations, including dietary supplements (DSTB1–DSTB3) and a pharmaceutical drug product (PDTB1). The vitamin D_3_ peak was identified based on retention time and UV absorption spectrum matching those of a reference standard. Panels A–D represent four repeated injections of each sample to illustrate chromatographic repeatability.

The variation in peak sizes observed for the tablets is due to the use of smaller tablet fragments (Figure 9). This approach was chosen to facilitate easier processing and handling.

The measured concentrations of vitamin D_3_ in the supplements ranged from 40 to 100 µg per serving (Table 3). All four products contained vitamin D_3_ levels within the regulatory tolerance limits (−20% to +50%) defined for dietary supplements [19], with measured values ranging between 94% and 108% of the labeled content.

## 3. Discussion

### 3.1. Questionnaire

Our survey aimed to assess the public knowledge and behaviors regarding vitamin D_3_ supplementation among 367 participants. The results indicated a general lack of knowledge about dietary supplementation, with many participants unaware of what a dietary supplement is, despite using them regularly. Only a small proportion of respondents (3.5%) answered all knowledge-based questions correctly, which may reflect common misconceptions regarding dietary supplements and the widespread perception that they are inherently beneficial and safe. This finding aligns with previous studies that have documented similar knowledge gaps in the population, underscoring the need for improved public health messaging [20,21]. The observed lack of knowledge surrounding supplementation highlights the necessity for targeted educational campaigns aimed at enhancing public understanding. Healthcare providers should be equipped with resources to educate their patients about the importance of vitamin D_3_, both from dietary sources and supplements.

When asked about the source of recommendation for vitamin D_3_ supplementation, 58.3% of respondents cited physicians, 37.1% cited relatives, and only 24.8% of participants cited pharmacists. While it is essential that healthcare professionals play a central role, the influence of non-professional sources such as relatives highlights a potential concern. Advice from family members may often lack scientific basis and could lead to inappropriate dosing or misconceptions about supplementation. The relatively lower votes for pharmacists are also noteworthy, given their accessibility and expertise in medication and supplement counseling. As highly accessible healthcare professionals, pharmacists often represent the first point of contact for patients, providing not only medication dispensing but also counseling, monitoring, and education. Consequently, their contribution to optimizing therapy outcomes and supporting public health initiatives has become more recognized and valued than ever before [22,23,24].

Despite patients most frequently interacting with pharmacists within the healthcare system, only a small proportion of respondents (8.4%) reported that pharmacists inquire about other dietary supplements during medication dispensing, while the majority (72.8%) said no and 18.8% could not remember. These findings underscore the need to strengthen pharmaceutical care in Hungary to reduce the risk of unwanted supplement-drug interactions and adverse effects. As shown in a Hungarian community pharmacy study [25], proactive inquiry about patients’ supplement use could identify potential interactions and improve safety. Encouraging pharmacists to actively inquire about all medications and supplements could improve the safe and rational use of dietary supplements. Unfortunately, physicians are often unaware of other supplements their patients are taking [26], which can lead to interactions resulting in severe side effects.

The responses regarding the timing of vitamin D_3_ supplementation highlight considerable variability in attitudes and practices. While a substantial proportion of participants reported taking vitamin D_3_ throughout the year (38.4%), nearly as many limited supplementation to the winter months (36.5%). This seasonal pattern likely reflects the common perception that supplementation is only necessary when sun exposure is reduced. However, given the high prevalence of vitamin D_3_ deficiency in Central and Eastern Europe [8], year-round supplementation would often be more appropriate. A smaller proportion of respondents reported taking vitamin D_3_ only when ill (3%) or sporadically when they remembered (11.7%), indicating inconsistent use and a lack of awareness that adequate vitamin D_3_ status requires regular intake. Notably, 10.4% of participants reported not taking vitamin D_3_ at all, which is concerning in light of the generally low dietary intake observed in Hungary [27].

On the question “How confident are you that the vitamin D_3_ product you are taking is effective?” most respondents (56%) reported absolute confidence, 41.9% were unsure, and 2.1% expressed no confidence. This uncertainty may reflect limited awareness of product regulation and quality standards or prior inconsistent experiences. Efforts should be made to increase patients’ motivation to ask questions about the products they use and to ensure they obtain information only from reliable sources.

Overall, this survey highlights a critical need for increased awareness and education regarding vitamin D_3_ supplementation, which could ultimately lead to improved health outcomes.

### 3.2. HPLC Analysis

Our HPLC analysis showed that all tested supplements were within acceptable variance limits as defined by regulatory standards [18]. The actual vitamin D_3_ content ranged from 89% to 117% of the labeled amounts, indicating general compliance with label claims (Table 3). In contrast to our findings of close label compliance, other studies have reported notable differences. In a study of marketed vitamin D_3_ formulations, only 60% of products fell within ±10% of their labeled vitamin D_3_ content, and non-prescription dietary supplements showed a wide range of actual content (8% to 201% of label claim), underscoring variability in supplement accuracy by product and manufacturer [28]. Vitamin D_3_ is relatively cheap and difficult to overdose on, leading manufacturers to include higher amounts in a product rather than underdosing [29]. When looking at dietary supplements in general, even greater discrepancies can often be observed [30]. Dietary supplements are often found to contain ingredients that are not listed on the packaging [31]. Conversely, there are also instances where a listed ingredient is entirely absent from the product [32].

We found that short-term daylight exposure did not drastically reduce vitamin D_3_ content in most products (only one supplement showed ~15% loss), suggesting that typical storage conditions are unlikely to degrade potency quickly. However, extended or improper storage might have greater effects [33].

Our HPLC method proved to be robust and sensitive, with high accuracy. It provides a reliable approach for future studies exploring broader classes of supplements and enhancing quality assurance practices. Ensuring accurate labeling is crucial for maintaining consumer trust and preventing health risks associated with vitamin D_3_ deficiency or excess. By providing a reliable and accurate means to quantify the actual vitamin D_3_ content in supplements, the method helps ensure that manufacturers comply with regulatory requirements and accurately label their products. This is particularly important, as deviations between labeled and actual vitamin D_3_ content are frequently reported in the literature, with discrepancies sometimes exceeding 50–80% [34]. Through routine testing, authorities can identify non-compliant products, safeguarding consumer health and promoting trust in the dietary supplement industry. Moreover, the method’s sensitivity and precision allow detection of potential over- or under-dosages, contributing to both consumer safety and product efficacy.

Overall, this HPLC method supports quality control and market transparency for various compositions of vitamin D_3_ supplement products. We provide a detailed workflow for analyzing both oil-based gel capsules and solid tablets, which can aid other laboratories in similar quality assessments.

### 3.3. Limitations

Regarding the survey, the data were self-reported, which may introduce recall or social desirability bias. The sample was predominantly young and highly educated, limiting the generalizability of the findings to the broader population. Future studies should include a more diverse population to better understand the factors contributing to knowledge gaps and to evaluate the effectiveness of targeted interventions.

Concerning the HPLC and stability analyses, only five capsules and four tablets were tested, limiting the generalizability of the results to all commercially available supplements. The daylight exposure stability study lasted only one month and under controlled laboratory conditions, which may not fully reflect real-life storage or environmental conditions, such as prolonged sunlight exposure, temperature fluctuations, or varying humidity.

HPLC measurements inherently involve analytical uncertainty, which may slightly affect the accuracy of the determined vitamin D_3_ content. Future studies should consider longer-term stability testing under various environmental conditions and include a larger number of products to provide more comprehensive insights.

### 3.4. Strengths

The survey included a relatively large sample of 367 respondents, which increases the statistical power and reliability of the findings. Importantly, the data were collected in the post–COVID-19 period, providing timely and up-to-date insight into vitamin D_3_-related knowledge and supplement use following a phase of markedly increased public and clinical attention to vitamin D_3_ supplementation.

The questionnaire development was based on previous literature and the authors’ professional experience, ensuring internal consistency, relevance, and coverage of multiple aspects of vitamin D_3_ knowledge and supplement use, thereby offering a comprehensive overview. Notably, the study contributes valuable data on dietary supplement habits among younger adults, a population for which detailed and recent evidence remains limited in the existing literature.

Regarding the analytical component, the HPLC method was fully validated, demonstrating excellent linearity, appropriate analytical range, accuracy, and precision, with well-determined LOD and LOQ, ensuring reliable and reproducible measurement of vitamin D_3_ content. No interfering peaks were observed around the vitamin D_3_ retention time, indicating the method’s suitability for complex supplement matrices.

Additionally, the stability study, although limited to one month of daylight exposure, provides preliminary insight into the robustness of vitamin D_3_-containing products under environmentally relevant light conditions. Taken together, a notable strength of this study is its dual approach, integrating public survey data with laboratory quality analysis. This provides a more holistic understanding of the vitamin D_3_ supplement landscape, from consumer knowledge to product content.

## 4. Materials and Methods

### 4.1. Materials

Vitamin D_3_ standard (Sigma-Aldrich, Budapest, Hungary, Lot number: 0000309709) was purchased from Merck on the 10 April 2024. Vitamin D_3_ dietary supplements, including soft gel capsules (DSGC1–DSGC4) and tablets (DSTB1–DSTB3), as well as one pharmaceutical drug product (PDTB1), were purchased from a community pharmacy (Pannon Pharmacy, Pécs, Hungary). One dietary supplement (DSGC5) was obtained from an online retailer [19]. Product codes were used to anonymize manufacturers; detailed product characteristics, including brand names, excipients, expiration dates, and storage conditions, are provided in Table S1 (Supporting Information).

The survey (Files S1 and S2) advertised via the university newsletter and Facebook assessed usage patterns of vitamin D_3_ supplements. Based on responses, the most frequently used supplements were selected. Due to product unavailability, the number of tablet supplements included in the final analysis was reduced from five to four.

### 4.2. Methods

#### 4.2.1. Survey Design

##### Study Design and Participants

The primary reason for designing this questionnaire was to determine which vitamin D_3_ supplements are most popular among consumers. Participants have named 64 different products and we selected the top five brands with the highest votes and analyzed their content. Additionally, to include a product from a bodybuilding website, we purchased one from Gymbeam for further analysis. This study employed a cross-sectional design using an anonymous, self-administered online questionnaire. Participants were recruited between 1 May 2024 and 30 June 2024 through the university newsletter and Facebook, targeting students, staff and the general public. Inclusion criteria were age ≥ 18 years, fluency in Hungarian or English and access to the internet.

##### Questionnaire Development

The questionnaire was developed based on a review of existing literature on supplements and dietary supplement use [35,36,37]. It consisted of 39 questions divided into sections covering general demographic information, knowledge about dietary supplements, knowledge and purchasing habits related to vitamin D_3_-containing dietary supplements, information regarding medication use. The questionnaire was prepared in Hungarian and translated into English; both language versions are provided as supplementary files (S1 and S2 files). The final version was administered via Google Forms, and responses were collected anonymously. Participation was voluntary, and respondents were informed about the purpose of the study, estimated completion time, and their right to withdraw at any time without any consequences.

##### Data Collection and Storage

Data were collected between 1 May 2024 and 30 June 2024 and stored securely on password-protected servers in compliance with data protection regulations. No personally identifiable information was collected.

##### Ethical Considerations

Ethical approval was not required for this study, as the questionnaire was anonymous and participation was voluntary, with no sensitive personal data collected. The survey complied with general data protection regulation (GDPR) requirements.

##### Statistical Analysis

The statistical analysis of the questionnaire data was primarily descriptive in nature. The objective of the survey was exploratory, aiming to characterize knowledge levels, usage patterns, and consumer preferences rather than to test predefined hypotheses. Therefore, inferential statistical analyses were not performed. Frequencies, percentages, and mean ± standard deviation were considered sufficient to describe the observed trends within the study population.

#### 4.2.2. HPLC Method

The HPLC method used for the determination of vitamin D_3_ was adapted from previously published analytical procedures for the quantification of vitamin D compounds using HPLC-UV detection, with minor modifications to the sample preparation and chromatographic conditions to accommodate the matrices of commercial dietary supplements [14].

##### Instrumentation

The qualitative and quantitative analysis of vitamin D_3_ in the dietary supplement samples were performed using a Jasco HPLC system (Jasco, Tokyo, Japan) equipped with a PU-4180 binary pump, AS-4050 autosampler, and UV-4070 detector (Jasco, Tokyo, Japan). The analysis was carried out on a Kinetex EVO C18 column (Phenomenex, 250 × 4.6 mm, 5 µm) coupled with a Phenomenex Security Guard column (Phenomenex, Torrance, CA, USA; C18, 4.0 × 3.0 mm). Data acquisition and evaluation were carried out using ChromNav Ver.2 software.

##### Type of Elution

An isocratic elution was applied, using a mixture of methanol (Molar Chemicals Kft.-Halásztelek, Hungary) and ultrapure water (ICW-3000™ Water Purification System, © 2024 Merck KGaA, Darmstadt, Germany) (95:5, *v*/*v*), optimized to achieve efficient separation of vitamin D_3_ from other matrix components. The injected sample volume was 20 µL, and the flow rate was maintained at 1.0 mL/min throughout the analysis. Vitamin D_3_ was detected at 265 nm.

##### Quantitative and Qualitative Analysis

Qualitative identification of vitamin D_3_ was performed by comparing the retention time and UV absorption spectrum of sample peaks with those of a certified vitamin D_3_ reference standard. Quantitative analysis was based on calibration with standard solutions of vitamin D_3_. A linear calibration curve was constructed to determine the concentration of vitamin D_3_ in the samples. The method was validated for linearity, accuracy, and reproducibility, ensuring reliable and precise quantification.

For quantitative analysis, the supplements were classified as compliant with their labeled vitamin D_3_ content if the measured concentration fell within the upper (+50%) and lower (−20%) limits defined by the EU guidance on tolerances for nutrient values [18]. Additionally, the vitamin D_3_ content of the capsules was re-measured after one month of storage, during which the blister packs were deliberately exposed to direct sunlight to assess potential degradation or any stability issues.

#### 4.2.3. Sample Preparation

##### Standard Solutions

Every day, before the HPLC measurements, a fresh, concentrated vitamin D_3_ stock solution (1.0 mg/mL) was prepared in olive oil (Sigma-Aldrich, CAS number: 8001-25-0) for the soft gelatin capsules and in isopropanol (Molar Chemicals Kft.-Halásztelek, Hungary) for the tablets, from which a standard dilution series was created using the respective solvent mixtures of the sample preparation techniques (see below). The standard series consisted of the following concentrations: 0.1, 0.05, 0.01, and 0.005 mg/mL. Based on the standard series, a calibration curve was established using Excel, which allowed us to determine the concentration of our samples.

##### Soft Gelatin Capsules

Soft gel capsules were frozen at −80 °C for 24 h and the outer gelatin protective layer was carefully removed thereafter. The oily solution from the inside of the capsule was weighed and its volume was determined accurately. Then, 1% (*v*/*v*) ascorbic acid (Sigma-Aldrich, Budapest, Hungary) dissolved in ultrapure water was added to the liquid content of the capsules, followed by saponification with an equal volume of 10 mM potassium hydroxide (KOH, Sigma-Aldrich, Budapest, Hungary). The mixture was vortexed thoroughly and diluted fivefold with isopropanol. The sample was subjected to sonication (ultrasonic water bath) for 3 min, followed by centrifugation at 6000× *g* for 5 min at 24 °C. The supernatant was directly injected into the HPLC system for analysis.

##### Tablets

Two different sample preparation methods were applied for the analysis of vitamin D_3_ in tablets due to differences in excipient composition among the products. Certain tablet formulations required an acidic aqueous buffer to effectively extract vitamin D_3_ [38], while others necessitated the use of an organic solvent (isopropanol) in combination with water to achieve complete dissolution of the active compound [39]. The choice of extraction method for each tablet ensured maximal recovery of vitamin D_3_ prior to HPLC analysis.

Method I: First, the tablets were weighed and an equivalent mass of hydrochloric acid buffer (Molar Chemicals Kft.-Halásztelek, Hungary) (pH = 3) was added. The mixture was then diluted with ultrapure water to a final volume of 1.8 mL. Ultrasonic treatment was performed for 10 min in water bath, followed by incubation in a water bath for 20 min at 37 °C. After processing, the sample was centrifuged at 6000× *g* for 5 min at 24 °C. The resulting supernatant was directly injected into the HPLC system for analysis.

Method II: The tablet was weighed and 1.8 mL of water was added. The mixture underwent ultrasonic treatment for 10 min, after which 1.8 mL of isopropanol (IPA) was added. The sample was then incubated in a water bath (at 37 °C) for 20 min. Following this, the mixture was centrifuged at 6000× *g* for 5 min at 24 °C. The supernatant was directly injected into the HPLC system for analysis.

#### 4.2.4. Testing the Stability of the Preparations Under UV Light

We also investigated the effect of natural daylight exposure on the stability of vitamin D_3_. For this purpose, four selected vitamin D_3_ supplement products (DSGC1-4) were subjected to a light-exposure experiment between 20 September and 21 October 2024 (Pécs, Hungary, 46.0713° N, 18.2331° E). To simulate potential degradation under natural light conditions, blister packs of each capsule product were placed near a south-facing laboratory window, allowing exposure to ambient daylight over a one-month period.

During the exposure period, environmental conditions, including room temperature and relative humidity, were monitored and remained stable. The average daily duration of sunshine during the study period was approximately 4–5 h per day. Following the one-month exposure, the vitamin D_3_ content of the preparations was re-evaluated using HPLC.

#### 4.2.5. Method Validation

The HPLC method was validated according to standard analytical performance requirements. Linearity and range were assessed by analyzing five calibration levels covering the expected concentration interval of vitamin D_3_; calibration curves were constructed using peak area versus concentration and linearity was evaluated by the coefficient of determination (R^2^) and residual analysis. LOD and LOQ were established based on signal-to-noise ratios of approximately 3:1 and 10:1, respectively. Repeatability (intra-day precision) was determined by analyzing replicate sample solutions (*n* = 3) at a single concentration level and calculating the RSD. System suitability was evaluated at the beginning of each analytical batch by performing eight consecutive injections of the same standard solution.

## 5. Conclusions

The present study combined a public survey with the analytical evaluation of vitamin D_3_ supplements marketed in Hungary. While the tested products generally demonstrated acceptable accuracy in relation to their labeled vitamin D_3_ content, the survey results revealed substantial gaps in consumer knowledge regarding dietary supplements. These findings highlight the importance of improved patient education and counseling by healthcare professionals. Furthermore, although the measured values fell within accepted regulatory limits, the variability observed in some products and the potential influence of environmental factors such as light exposure emphasize the need for continued quality monitoring of dietary supplements available on the market.

## Figures and Tables

**Table 1:** Demographic characteristics of the participants.

Variable	Category	*n*	%
Sex	Female	251	60%
	Male	116	40%
Age	Mean ± SD	31.36 ± 12.48	–
County of Residence	Baranya	179	48.8%
	Pest	62	16.9%
	Tolna	21	5.7%
	Zala	16	4.4%
Place of Residence	Capital city	44	12%
	County town	171	46.6%
	City	57	15.5%
	Small town	23	6.3%
	Large village	7	1.9%
	Village	24	6.5%
	Hamlet	41	11.2%
Highest Education	Primary School	4	1.1%
	Vocational Secondary School	9	2.5%
	Secondary School Diploma	158	43.1%
	High School or University	183	49.9%
	PhD/DLA	13	3.5%

Sex, place of residence, county of residence, and highest educational level are presented as absolute numbers (*n*) and percentages (%). Age is reported as mean ± standard deviation (SD). Percentages may not sum to 100% due to rounding.

**Table 2:** True/false statements regarding dietary supplements.

Statement	True	False	I Do Not Know
Dietary supplements are also considered food products.	**32.4%**	55.9%	11.7%
The effectiveness of dietary supplements is not supported by clinical trials.	**22.6%**	65.9%	11.4%
Dietary supplements do not require pre-market approval; their sale only needs to be reported.	**28.6%**	62.7%	8.7%
Dietary supplements play an important role in disease prevention and treatment.	70.8%	**16.3%**	12.8%
If we eat a balanced diet, there is no need for dietary supplements.	**48.2%**	42.5%	9.3%

Correct answers were determined based on current regulatory definitions of dietary supplements and evidence-based clinical guidelines regarding their use [17]. The correct answers are highlighted in bold.

**Table 3:** Comparison of nominal and actual vitamin D_3_ content in preparations.

Code	Date of Analysis	Labeled Dose (µg)	Acceptable Dose Range (µg)	Measured Amount Per Serving (µg)	% of Labeled Dose	SD
DSGC1	17 September 2024	50	40–75	58.53	117.07	1.08
DSGC2	16 September 2024	50	40–75	44.72	89.45	0.19
DSGC3	16 September 2024	75	60–112.5	68.74	91.65	1.00
DSGC4	17 September 2024	55	44–82.5	57.98	105.41	0.80
DSGC5	23 September 2024	50	40–75	51.56	103.12	1.01
DSTB1	14 November 2024	100	80–150	108.70	108.70	1.56
DSTB2	15 November 2024	75	60–112.5	73.79	98.38	4.25
DSTB3	20 December 2024	100	80–150	94.46	94.46	5.96
PDTB1	14 November 2024	40	32–60	43.44	108.59	3.33

Labeled dose, acceptable dose range, and measured vitamin D_3_ content per serving are expressed in micrograms (µg). Measured values represent the mean ± standard deviation (SD) of replicate analyses (*n* = 4). The percentage of labeled dose was calculated as the ratio of measured to labeled vitamin D_3_ content. Abbreviations: DSGC—dietary supplement gel capsule; DSTB—dietary supplement tablet; PDTB—pharmaceutical drug tablet. Numeric suffixes indicate individual products.

**Table 4:** Comparison of nominal and actual vitamin D_3_ content in preparations after one month of daylight exposure.

Code	Date of Analysis	Labeled Dose (µg)	Acceptable Dose Range (µg)	Measured Amount Per Serving (µg)	% of Labeled Dose	SD
DSGC1	28 October 2024	50	40–75	57.04	114.08	0.10
DSGC2	28 October 2024	50	40–75	**38.16**	**76.32**	13.14
DSGC3	28 October 2024	75	60–112.5	77.29	103.05	0.45
DSGC4	28 October 2024	55	44–82.5	65.42	118.94	1.25

Vitamin D_3_ content was determined by HPLC and expressed as micrograms (µg) per serving. Results are presented as mean ± standard deviation (SD). The percentage of labeled dose was calculated relative to the declared vitamin D_3_ content. Significant decreases in vitamin D_3_ content after daylight exposure are highlighted in bold. DSGC—dietary supplement gel capsule.

## Data Availability

The data presented in this study are available from the corresponding author upon reasonable request.

## Supplementary Materials

The following supporting information can be downloaded at: https://www.mdpi.com/article/10.3390/ph19030493/s1, Figure S1. Determination of the limit of detection (LOD) for vitamin D_3_ by RP-HPLC; Figure S2. Determination of the limit of quantification (LOQ) for vitamin D_3_ by RP-HPLC; Figure S3. Repeatability of the method using a 0.05 mg/mL vitamin D_3_ standard; File S1. Hungarian version of the vitamin D_3_ supplementation questionnaire; File S2. English version of the vitamin D_3_ supplementation questionnaire; Table S1. Characteristics of the vitamin D_3_ dietary supplements and pharmaceutical products included in the analytical study.

## Abbreviations

The following abbreviations are used in this manuscript:

As	Symmetry Factor
AUC	Area Under the Curve
CVD	Cardiovascular Disease
DSGC	Dietary Supplement Gel Capsule
DSTB	Dietary Supplement Tablet
GDPR	General Data Protection Regulation
HPLC	High-Performance Liquid Chromatography
HPLC-UV	High-Performance Liquid Chromatography with Ultraviolet Detection
IPA	Isopropanol
KOH	Potassium Hydroxide
LOD	Limit of Detection
LOQ	Limit of Quantification
µg	Microgram
n	Absolute Number
PDTB	Pharmaceutical Drug Tablet
%	Percentage
R^2^	Coefficient of Determination
RSD	Relative Standard Deviation
SD	Standard Deviation
UVB	Ultraviolet B
VDR	Vitamin D Receptor
25(OH)D	25-Hydroxyvitamin D
1,25(OH)_2_D	1,25-Dihydroxyvitamin D
