# Supplementary material for: Quantitative Analysis of the Vitamin D3 Content in Dietary Supplements Marketed in Hungary Using High-Performance Liquid Chromatography
Source: Pharmaceuticals (Basel). 2026 Mar 17;19(3):493. doi: 10.3390/ph19030493 (PMC13028880; doi:10.3390/ph19030493)
Supplement: Supplementary file 1 [file pharmaceuticals-19-00493-s001.zip › S1 File.pdf]

A lakosság D-vitamin tartalmú étrend-kiegészítő alkalmazásának online felmérése

Kedves Érdeklődő!

Az alábbi kérdőívet a Pécsi Tudományegyetem Gyógyszerésztudományi Kar Gyógyszerhatástani Tanszék és a Gyógyszerészeti Intézet gyógyszerész oktató munkatársai állították össze a D-vitamin tartalmú étrend-kiegészítők népszerűségének megismerése céljából.

Az étrend-kiegészítők a kiegyensúlyozott, vegyes étrend kiegészítését szolgáló élelmiszerek, amelyek koncentrált formában tartalmaznak tápanyagokat vagy egyéb táplálkozási vagy élettani hatással rendelkező anyagokat, egyenként vagy kombináltan és adagolható formában (kapszula, tabletta, adagolható por, adagolható folyadék stb.) kerülnek forgalomba.

Kérdőíves megkérdezésünk során arra keressük a választ, hogy jelenleg mely készítmények a legnépszerűbbek, milyen ismeretekkel rendelkeznek az étrend-kiegészítő terméket fogyasztók és miért választanak egy-egy adott terméket. A felmérés eredményeit a későbbi próbavásárlásokhoz és analitikai vizsgálatokhoz fogjuk felhasználni, mely során különböző D-vitamin tartalmú készítmények beltartalmát fogjuk vizsgálni.

A válaszadás önkéntes és semmilyen következménnyel nem jár a kitöltőre nézve. A résztvevő bármikor visszavonhatja válaszait, ehhez a vizsgálatért felelős kollégával kell felvennie a kapcsolatot. A vizsgálat során gyűjtött adatokat szigorúan bizalmasan kezeljük és anonimizált módon tudományos kutatási célú elemzések készítésére használjuk fel. A vizsgálat eredményei kizárólag olyan formában kerülnek közlésre, amely a résztvevők személyének azonosítására alkalmas személyes adatokat (név, születési dátum stb.) nem tartalmazzák. Ha Önnek kérdése van a vizsgálattal kapcsolatban vagy további információkat szeretne kapni, akkor az alábbi, vizsgálatért felelős személlyel veheti fel a kapcsolatot:

Dr. Nagy András, PTE GYTK Gyógyszerhatástani Tanszék, Központi Rezidens, 7624 Pécs, Rókus utca 2., telefon: 28317, email: nagy.andras@pte.hu

A kitöltés ideje 5-10 perc

* Kötelező kérdés

I. Általános demográfiai kérdések

1. Neme *

Soronként csak egy oválist jelöljön be.

- Nő
- Férfi
- Egyéb/ Nem kíván válaszolni

2. Kora *

____

3. Melyik vármegyében van a lakóhelye? *

Soronként csak egy oválist jelöljön be.

- Baranya
- Bács-Kiskun
- Békés
- Borsod-Abaúj-Zemplén
- Csongrád-Csanád
- Fejér
- Győr-Moson-Sopron
- Hajdú-Bihar
- Heves
- Jász-Nagykun-Szolnok
- Komárom-Esztergom
- Nógrád
- Pest
- Somogy
- Szabolcs-Szatmár-Bereg
- Tolna
- Vas
- Veszprém
- Zala
- Külföldön lakom

4. Az ön lakóhelyének jellege *

Soronként csak egy oválist jelöljön be.

- Főváros
- Megyei jogú város
- Város
- Kisváros
- Nagyközség
- Község
- Falu

5. Legmagasabb iskolai végzettség *

Soronként csak egy oválist jelöljön be.

- Általános Iskola
- Szakközépiskola
- Érettségi
- Főiskola vagy Egyetem
- PhD/DLA

II. Étrend-kiegészítőkkel kapcsolatos ismeretek felmérése

Ezen az oldalon többek között igaz-hamis állításokkal szeretnénk felmérni, hogy milyen ismeretekkel rendelkeznek az étrend-kiegészítő terméket fogyasztók és miért választanak egy-egy adott terméket. Kérjük saját tudása szerint válaszoljon!

6. Az étrend-kiegészítők is élelmiszerek. *

Soronként csak egy oválist jelöljön be.

- Igaz
- Hamis
- Nem tudom megítélni

7. Az étrendkiegészítők hatékonyságát nem támasztják alá klinikai vizsgálatokkal. *

Soronként csak egy oválist jelöljön be.

- Igaz
- Hamis
- Nem tudom megítélni

8. Az étrend-kiegészítőket forgalmazás előtt nem kell engedélyeztetni, csak a termék forgalmazását kell bejelenteni. *

Soronként csak egy oválist jelöljön be.

- Igaz
- Hamis
- Nem tudom megítélni

9. Az étrend-kiegészítők fontos szerepet játszanak a betegségek megelőzésében és gyógyításában. *

Soronként csak egy oválist jelöljön be.

- Igaz
- Hamis
- Nem tudom megítélni

10. Ha kiegyensúlyozottan táplálkozunk, akkor nincs szükségünk étrend-kiegészítőkre. *

Soronként csak egy oválist jelöljön be.

- Igaz
- Hamis
- Nem tudom megítélni

11. Az étrendkiegészítőknek mit kell feltüntetni az alábbiak közül? *

Válassza ki az összeset, amely érvényes.

- [ ] A termék ajánlott napi fogyasztási mennyiségét
- [ ] Figyelmeztetést, hogy a fogyasztó ne vegyen be az ajánlott napi adagnál többet
- [ ] Nyilatkozatot arról, hogy az étrend-kiegészítők nem helyettesítik a kiegyensúlyozott táplálkozást
- [ ] Figyelmeztetést arra, hogy a termékeket kisgyermekektől elzárva kell tárolni
- [ ] Egyik sem

12. Hallott-e étrend-kiegészítő hamisításról? *

Soronként csak egy oválist jelöljön be.

- Igen
- Nem

13. Az alábbi skálán jelölje, hogy melyik forrást mennyire tartja biztonságosnak az étrend-kiegészítők beszerzésével kapcsolatban! *

Jelölje az alábbi 1-től 5-ig terjedő skálán, ahol 1 az „egyáltalán nem biztonságos”-t, míg az 5 „Teljes mértékben biztonságos”-t jelöli.

Soronként csak egy oválist jelöljön be.

	1	2	3	4	5
**Gyógyszertár**					
**Online gyógyszertár**					
**Gyógyszertáron kívüli forgalmazás (benzinkút, drogéria, élelmiszerbolt)**					
**Egyéb internetes felület**					

14. Az alábbiak közül kinek a tanácsát fogadná el egy étrend-kiegészítő alkalmazásával kapcsolatosan? *

Válassza ki az összeset, amely érvényes.

- [ ] Gyártó
- [ ] Orvos
- [ ] Gyógyszerész
- [ ] Rokon
- [ ] Barát
- [ ] Szomszéd
- [ ] Egyéb: ______________________

15. Hányszor használta már az internetet étrend-kiegészítő vásárlásra?

Soronként csak egy oválist jelöljön be.

- ( ) Soha
- ( ) Vásároltam már egy-két alkalommal
- ( ) Több alkalommal vásároltam már
- ( ) Rendszeresen vásárolok

16. Az elmúlt egy évben mennyit költött étrend-kiegészítők vásárlására? *

Soronként csak egy oválist jelöljön be.

- ( ) Nem szedek étrend-kiegészítőt
- ( ) Nem vettem étrend-kiegészítőt (de szedek)
- ( ) 10000 ft alatt
- ( ) 10-20000 ft között
- ( ) 20-50000 ft
- ( ) 50-100000 ft
- ( ) 100000 ft felett

17. Mennyire gondolja magát tájékozottnak a szedett étrendkiegészítőjével kapcsolatban? *

Soronként csak egy oválist jelöljön be.

	1	2	3	4	5	
Egyá	◯	◯	◯	◯	◯	Teljes mértékben

III. D-vitamin tartalmú étrend-kiegészítőkkel kapcsolatos ismertek és vásárlási szokások felmérése

18. Mennyire tartja megfelelő beszerzési formának az alábbi lehetőségeket D-vitamin tartalmú étrend-kiegészítők tekintetében? *

Jelölje az alábbi 1-től 5-ig terjedő skálán, ahol 1 az „egyáltalán nem megfelelő"-t, míg az 5 „teljes mértékben megfelelő"-t jelöli.

Soronként csak egy oválist jelöljön be.

	1	2	3	4	5
**Gyógyszertár**	◯	◯	◯	◯	◯
**Online gyógyszertár**	◯	◯	◯	◯	◯
**Gyógyszertáron kívüli forgalmazás (benzinkút, drogéria, élelmiszerbolt)**	◯	◯	◯	◯	◯
**Egyéb internetes felület**	◯	◯	◯	◯	◯

19. Ki ajánlotta a D-vitamin tartalmú étrend-kiegészítő alkalmazását önnek? *

Válassza ki az összeset, amely érvényes.

- [ ] Orvos
- [ ] Gyógyszerész
- [ ] Asszisztens
- [ ] Természetgyógyász
- [ ] Ismerős
- [ ] Családtag
- [ ] Reklám
- [ ] Egyéb: ______________________

20. Milyen szempontok alapján választ magának D-vitamin tartalmú készítményt? *

Válassza ki az összeset, amely érvényes.

- [ ] Ár
- [ ] Ami épp akciós
- [ ] Márka
- [ ] Ajánlás szakembertől
- [ ] Ajánlás természetgyógyásztól
- [ ] Ajánlás ismerőstől
- [ ] TV Reklám
- [ ] Internetes reklám
- [ ] Egyéb: ______________________

21. Milyen célból alkalmazza a D-vitamin tartalmú étrend-kiegészítőt? *

Válassza ki az összeset, amely érvényes.

- [ ] Ne legyek beteg
- [ ] Általános egészségemhez kell
- [ ] Mert az orvos azt mondta
- [ ] Mert télen fokozott a D-vitamin szükséglet
- [ ] Nem szedek D-vitamin tartalmú étrend-kiegészítőt
- [ ] Egyéb: ______________________

22. Milyen formában preferálja leginkább a D-vitamin tartalmú étrend-kiegészítőt?

Soronként csak egy oválist jelöljön be.

- Tabletta
- Lágy kapszula
- Olajos csepp
- Mindegy, csak jó legyen

23. Mikor szed D-vitamin tartalmú étrend-kiegészítőt az év során? *

Soronként csak egy oválist jelöljön be.

- Egész évben
- Csak a téli időszakban
- Csak, amikor beteg vagyok
- Amikor éppen eszembe jut
- Nem szedek egyáltalán D vitamint

24. Mikor alkalmazza a D-vitamin tartalmú étrend-kiegészítőt a nap során?

Soronként csak egy oválist jelöljön be.

- Reggelente
- Ebédidőben
- Este
- Amikor éppen eszembe jut

25. Hol vásárol leggyakrabban D-vitamin tartalmú étrend-kiegészítőt? *

Soronként csak egy oválist jelöljön be.

- Gyógyszertár
- Online gyógyszertár
- Gyógyszertáron kívüli forgalmazó egységekből (benzinkút, drogéria, élelmiszerbolt)
- Egyéb internetes felületen
- Nem vásárlok D-vitamin tartalmú étrend-kiegészítőt
- Egyéb: ______________________

26. Konkrét forrás megnevezése:

____

27. Miért onnan szokott vásárolni?

____

28. Mennyire biztos abban, hogy az ön által szedett D-vitamin tartalmú étrend-kiegészítő biztonságos?

Soronként csak egy oválist jelöljön be.

- Teljes mértékben
- Nem vagyok benne biztos
- Egyáltalán nem

29. Mennyire biztos abban, hogy az ön által szedett D-vitamin tartalmú készítmény hatásos?

Soronként csak egy oválist jelöljön be.

- Teljes mértékben
- Nem vagyok benne biztos
- Egyáltalán nem

30. Tapasztalt bármiféle nem kívánt hatást a termék alkalmazása során?

Soronként csak egy oválist jelöljön be.

- Igen
- Nem

31. Ha igen, milyen nem kívánt hatást tapasztalt?

____

32. Gyakrabban vásárol olyan terméket, ami a D-vitamin mellett egyéb anyagot (pl. C-vitamin, K-vitamin, Kálcium, Vitaminok és nyomelemek) is tartalmaz? *

Soronként csak egy oválist jelöljön be.

- Igen
- Nem
- Talán
- Nem vásárlok D-vitamin tartalmú étrend-kiegészítőt

33. Mely D-vitamin tartalmú étrend-kiegészítőt szokta vásárolni? *

Válassza ki az összeset, amely érvényes.

- [ ] Vitaking D-2000 vitamin
- [ ] WeightWorld D3-vitamin
- [ ] NOW FOODS VITAMIN D
- [ ] Pharmekal D3-vitamin
- [ ] Natur Tanya Oliva D3-vitamin
- [ ] FutuNatura D3-vitamin
- [ ] Jutavit D3-vitamin
- [ ] Jutavit Multivitamin Immuner
- [ ] NATURLAND D-vitamin
- [ ] Eurovit D-vitamin
- [ ] Eurovit C+D vitamin
- [ ] Béres D3-vitamin
- [ ] BioCo D-vitamin
- [ ] BioCo C+D
- [ ] Biotech USA D3 vitamin
- [ ] One - A - Day Multivitamin Biotech USA
- [ ] C-vitamin + D3 GymBeam
- [ ] D3+K1+K2 vitamin - GymBeam
- [ ] Gym Beam vitamin D3
- [ ] Vitality Complex - GymBeam
- [ ] Nem vásárlok D-vitamin tartalmú étrend-kiegészítőt
- [ ] Egyéb: ______________________

34. Mennyire elégedett a szedett D-vitamin tartalmú étrend-kiegészítőjével?

Soronként csak egy oválist jelöljön be.

	1	2	3	4	5	
Egy	◯	◯	◯	◯	◯	Teljes mértékben

IV. Gyógyszerszedéssel kapcsolatos információk

35. Van krónikus betegsége? *

Soronként csak egy oválist jelöljön be.

- Igen
- Nem

36. Milyen krónikus betegsége van?

____

37. Hány gyógyszert szed?

____

38. Szed az orvos által felírt, vényköteles D-vitamint? *

Soronként csak egy oválist jelöljön be.

- Igen
- Nem

39. A gyógyszertárban rákérdeznek az egyéb szedett étrend-kiegészítőkre a gyógyszerkiváltás során? *

Soronként csak egy oválist jelöljön be.

- igen
- Nem
- Nem emlékszem

Köszönjük, hogy kitöltötte kérdőívünket!

40. Megjegyzések, észrevételek a kérdőívvel vagy témával kapcsolatban:

Amennyiben egészségügyi szakember/hallgató kérem itt jelezze!

____

Ezt a tartalmat nem a Google hozta létre, és nem is hagyta azt jóvá.

Google Űrlapok
