# Supplementary material for: Quantitative Analysis of the Vitamin D3 Content in Dietary Supplements Marketed in Hungary Using High-Performance Liquid Chromatography
Source: Pharmaceuticals (Basel). 2026 Mar 17;19(3):493. doi: 10.3390/ph19030493 (PMC13028880; doi:10.3390/ph19030493)
Supplement: Supplementary file 1 [file pharmaceuticals-19-00493-s001.zip › S1 Table.pdf]

Brand	Code	Pill type	Labeled Dose (ug)	Expiration	Type of excipient	Storage
Jutavit	DSGC1	soft gel	50	04.2026	olive oil	room temperature, away from direct sunlight
Béres Vita	DSGC2	soft gel	50	12.2025	olive oil	dry place, max 25 °C, away from direct sunlight
BioCo Oliva	DSGC3	soft gel	75	01.2026	olive oil	dry place, 15-25 °C, away from direct sunlight
Eurovit	DSGC4	soft gel	55	11.2025	olive oil	dry place, 15-25 °C, away from direct sunlight
Gymbeam	DSGC5	soft gel	50	02.2026	safflower oil	dry place, max 25 °C, away from direct sunlight
Jutavit Forte	DSTB1	tablet	100	07.2025		room temperature, away from direct sunlight
Eurovit Forte	DSTB2	tablet	75	11.2024		dry place, 15-25 °C, away from direct sunlight
Naturland	DSTB3	tablet	100	07.2026		dry place, max 25 °C, away from direct sunlight
Béres	PDTB1	tablet	40	11.2026		dry place, max 25 °C, away from direct sunlight

Table S1. Characteristics of the vitamin D_3 dietary supplements and pharmaceutical product included in the analytical study. The table summarizes product codes, dosage form, labeled vitamin D_3 content, expiration date, type of excipient, and manufacturer-recommended storage conditions. DSGC - dietary supplement gel capsule; DSTB - dietary supplement tablet; PDTB - pharmaceutical drug tablet; µg - micrograms. Numeric suffixes indicate individual products.
