# Supplementary material for: Quantitative Analysis of the Vitamin D3 Content in Dietary Supplements Marketed in Hungary Using High-Performance Liquid Chromatography
Source: Pharmaceuticals (Basel). 2026 Mar 17;19(3):493. doi: 10.3390/ph19030493 (PMC13028880; doi:10.3390/ph19030493)
Supplement: Supplementary file 1 [file pharmaceuticals-19-00493-s001.zip › S2 File.pdf]

Online Survey on the Use of Vitamin D Supplements in the Population

Dear Participant!

The following questionnaire was created by the Department of Pharmacology and the Institute of Pharmacy at the University of Pécs, Faculty of Pharmacy. It aims to gather information on the popularity and usage of vitamin D-containing dietary supplements.

Dietary supplements are foods designed to complement a balanced, varied diet. They contain concentrated nutrients or other substances with nutritional or physiological effects, either individually or in combination. These supplements are sold in forms like capsules, tablets, powders, or liquids, intended for ingestion in specific doses.

In our survey, we aim to find out which dietary supplements are currently the most popular, the knowledge possessed by consumers of these products, and why they choose particular ones. The results will later be used for pilot purchases and analytical tests, where we will examine the contents of various vitamin D containing supplements.

Participation in the survey is voluntary and has no consequences for the participant. Respondents can withdraw their answers at any time by contacting the person responsible for the study. Data collected during the study will be treated confidentially and used anonymously for scientific research. The results will only be presented in a way that does not allow the identification of participants. If you have any questions or need further information about the study, you can contact the responsible person listed below:

Dr. András Nagy, from the Department of Pharmacology, PTE Faculty of Pharmacy, is responsible for this survey. You can contact him via phone at 28317 or email at nagy.andras@pte.hu for any inquiries. The time needed to complete the survey is estimated at 5-10 minutes.

I. General demographic section

1. Sex:

- Female
- Male
- Other / Prefer not to answer

2. Age:

   ____

3. In Which county do you live?

- Baranya
- Bács-Kiskun
- Békés
- Borsod-Abaúj-Zemplén
- Csongrád-Csanád
- Fejér
- Győr-Moson-Sopron
- Hajdú-Bihar
- Heves
- Jász-Nagykun-Szolnok
- Komárom-Esztergom
- Nógrád
- Pest
- Somogy
- Szabolcs-Szatmár-Bereg
- Tolna
- Vas
- Veszprém
- Zala
- I live abroad

4. The type of your place of residence:

- Capital city
- County town
- City
- Small town
- Large village
- Village
- Hamlet

5. Highest Education

- Primary School
- Vocational Secondary School

- Secondary School Diploma
- High School or University
- PhD/DLA

II. Survey on Knowledge of Dietary Supplements

On this page, we aim to assess, through true-false statements, the knowledge of dietary supplement consumers and understand their reasons for choosing specific products. Please answer based on your own knowledge!

6. Dietary supplements are also considered food products.

- True
- False
- I don t know

7. The effectiveness of dietary supplements is not supported by clinical trials.

- True
- False
- I don t know

8. Dietary supplements do not require pre-market approval; their sale only needs to be reported.

- True
- False
- I don t know

9. Dietary supplements play an important role in disease prevention and treatment.

- True
- False
- I don t know

10. If we eat a balanced diet, there is no need for dietary supplements.

- True
- False
- I don t know

11. What information must dietary supplements include from the following options?

- The recommended daily intake of the product
- A warning not to exceed the recommended daily dose
- A statement that dietary supplements do not replace a balanced diet
- A warning to keep the product out of reach of children
- None of the above

12. Have you ever heard about dietary supplement counterfeiting?

- Yes
- No

13. On the following scale, please indicate how safe you consider each source for obtaining dietary supplements! Please mark on the 1 to 5 scale, where 1 means „not safe at all" and 5 means „completely safe".

	1	2	3	4	5
Pharmacy					
Online Pharmacy					
Sales outside of pharmacies (gas stations, grocery stores etc.)					
Other Online websites					

14. Who would you trust for advice regarding the use of dietary supplements from the following options?

- Manufacturer
- Physician
- Pharmacist
- Relative
- Friend
- Neighbour
- Other: ______________

15. How many times have you used the internet for purchasing dietary supplements?

- Never
- Rearly
- Few Times
- Regularly

16. How much have you spent on dietary supplements in the past year?

- I do not take supplements
- I haven t bought supplements (but I take)
- under 10000 HUF
- Between 10-20000 HUF
- Between 20-50000 HUF
- Between 50-100000 HUF
- More than 100000 HUF

17. How informed do you consider yourself regarding the dietary supplement you are taking (where 1 is „not informed at all” and 5 is „absolutely informed”)

- 1
- 2
- 3
- 4
- 5

III. III. Survey on Knowledge and Purchasing Habits Regarding Vitamin D Supplements

18. How appropriate do you consider the following methods for purchasing vitamin D supplements? Please mark on a scale from 1 to 5, where 1 means „not appropriate at all” and 5 means „completely appropriate”.

	1	2	3	4	5
Pharmacy					
Online Pharmacy					
Sales outside of pharmacies (gas stations, grocery stores etc.)					
Other Online websites					

19. Who recommended the use of vitamin D supplements to you?
- Physician
- Pharmacist
- Assistant
- Naturopath
- Friend
- Relative
- Advertisement

20. What factors do you consider when choosing a D-vitamin supplement?

- Price
- What is on sale
- Brand
- Recommendation from professional
- Recommendation from Naturopath
- Recommendation from friend
- Television advertisement
- Online advertisement

21. For what purpose do you use a D-vitamin supplement?

- To avoid being ill
- General health
- My doctor told me so
- Because in winter the need is higher
- I do not take dietary supplements containing vitamin D
- Other: ___________

22. What form do you prefer for a vitamin D supplement?

- Tablet
- Soft gel capsule
- drops
- Does not matter if it works

23. When do you take a D-vitamin supplement during the year?

- All year
- Only in winter
- When I am ill
- When I remember it
- I do not take dietary supplements containing vitamin D

24. When do you take the D-vitamin-containing dietary supplement during the day
    - Mornings
    - Lunchtime
    - Evenings
    - When I remember it

25. Where do you purchase vitamin D supplements the most?
    - Pharmacy
    - Online Pharmacy
    - Sales outside of pharmacies (gas stations, grocery stores etc.)
    - Other online websites
    - I do not purchase vitamin D supplements

26. Name the actual place please: ________________

27. Why do you buy from this place specifically? _______________

28. How confident are you that the vitamin D containing dietary supplement you are taking is safe?

- Absouletely confident
- I am not surely confident
- Not confident at all

29. How confident are you that the vitamin D containing product you are taking is effective?

- Absouletely confident
- I am not surely confident

- Not confident at all

30. Have you experienced any side effects during the treatment?

    - Yes
    - No

31. If yes, what have you experienced? ________________________

32. Do you more often buy products that contain other substances (e.g., Vitamin C, Vitamin K, Calcium, Vitamins, and trace elements) in addition to Vitamin D?

    - Yes
    - No
    - Maybe
    - I do not purchase vitamin D supplements

33. Which vitamin D supplements do you purchase?
    - Vitaking D-2000 vitamin
    - WeightWorld D3-vitamin
    - NOW FOODS VITAMIN D
    - Pharmekal D3-vitamin
    - Natur Tanya Oliva D3-vitamin
    - FutuNatura D3-vitamin
    - Jutavit D3-vitamin
    - Jutavit Multivitamin Immuner
    - NATURLAND D-vitamin
    - Eurovit D-vitamin
    - Eurovit C+D vitamin
    - Béres D3-vitamin
    - BioCo D-vitamin
    - BioCo C+D
    - Biotech USA D3 vitamin
    - One - A - Day Multivitamin Biotech USA
    - C-vitamin + D3 GymBeam
    - D3+K1+K2 vitamin - GymBeam
    - Gym Beam vitamin D3
    - Vitality Complex - GymBeam
    - I do not purchase vitamin D supplements

34. How satisfied are you with your vitamin D supplement (where 1 is „unsatisfied" and 5 is „very satisfied")?
    - 1
    - 2

- 3
- 4
- 5

IV. Information related to medication use

35. Do you have a chronic disease?

- Yes
- No

36. What kind of chronic disease do you have? ______________

37. How many medicine do you take? _______________

38. Are you taking prescriped vitamin D?

- Yes
- No

39. Do pharmacists inquire about other dietary supplements being taken during medication dispensing?

- Yes
- No
- I Do not remember

Thank you for filling our survey!

40. Comments or observations regarding the questionnaire or the topic: __________________
If you are a healthcare professional/student, please indicate here! ____________________
