# Supplementary figures and images for: Quantitative Analysis of the Vitamin D3 Content in Dietary Supplements Marketed in Hungary Using High-Performance Liquid Chromatography
Source: Pharmaceuticals (Basel). 2026 Mar 17;19(3):493. doi: 10.3390/ph19030493 (PMC13028880; doi:10.3390/ph19030493)

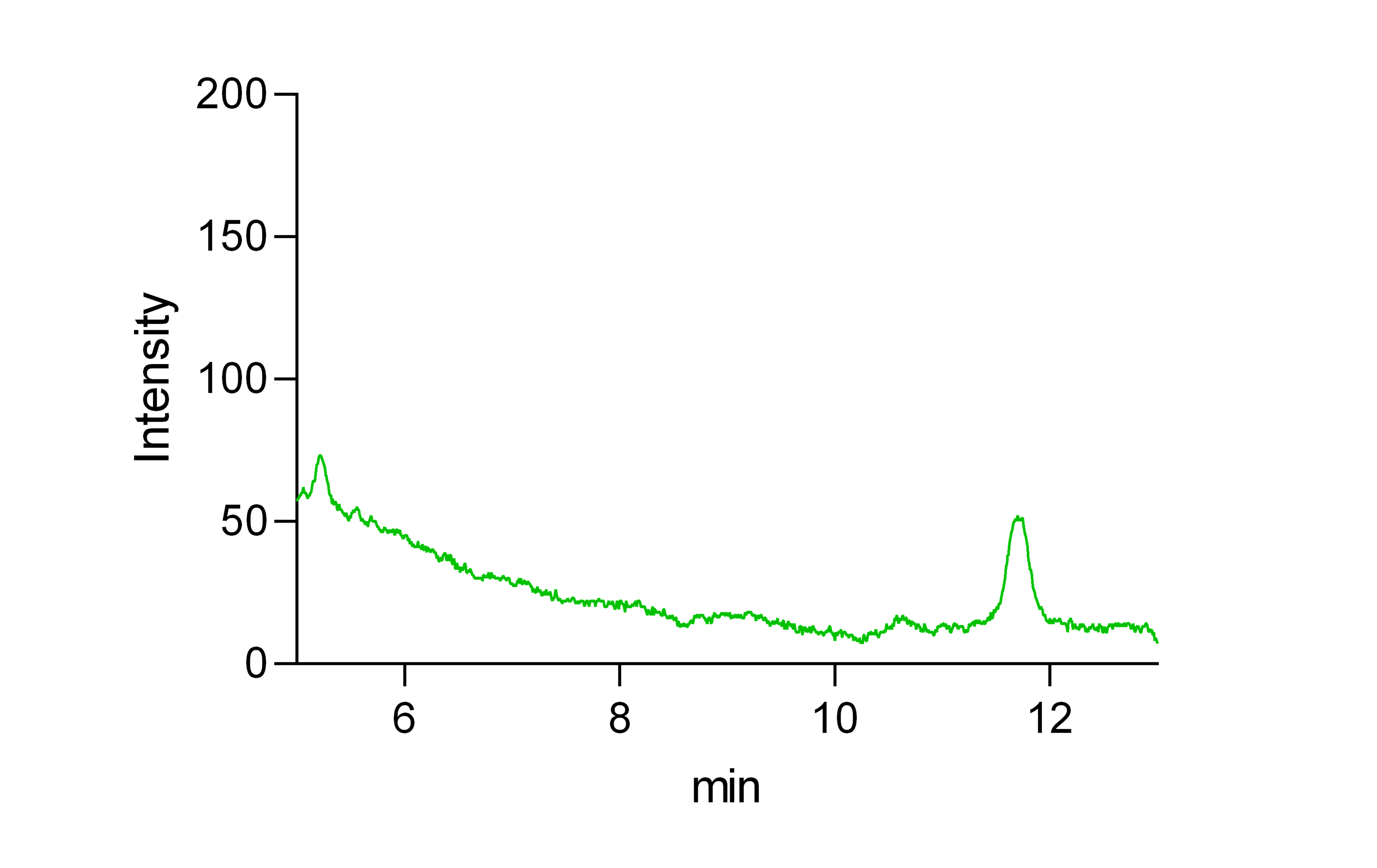

Supplement: Supplementary file 1 [file pharmaceuticals-19-00493-s001.zip › S2 Fig LOQ.tif]

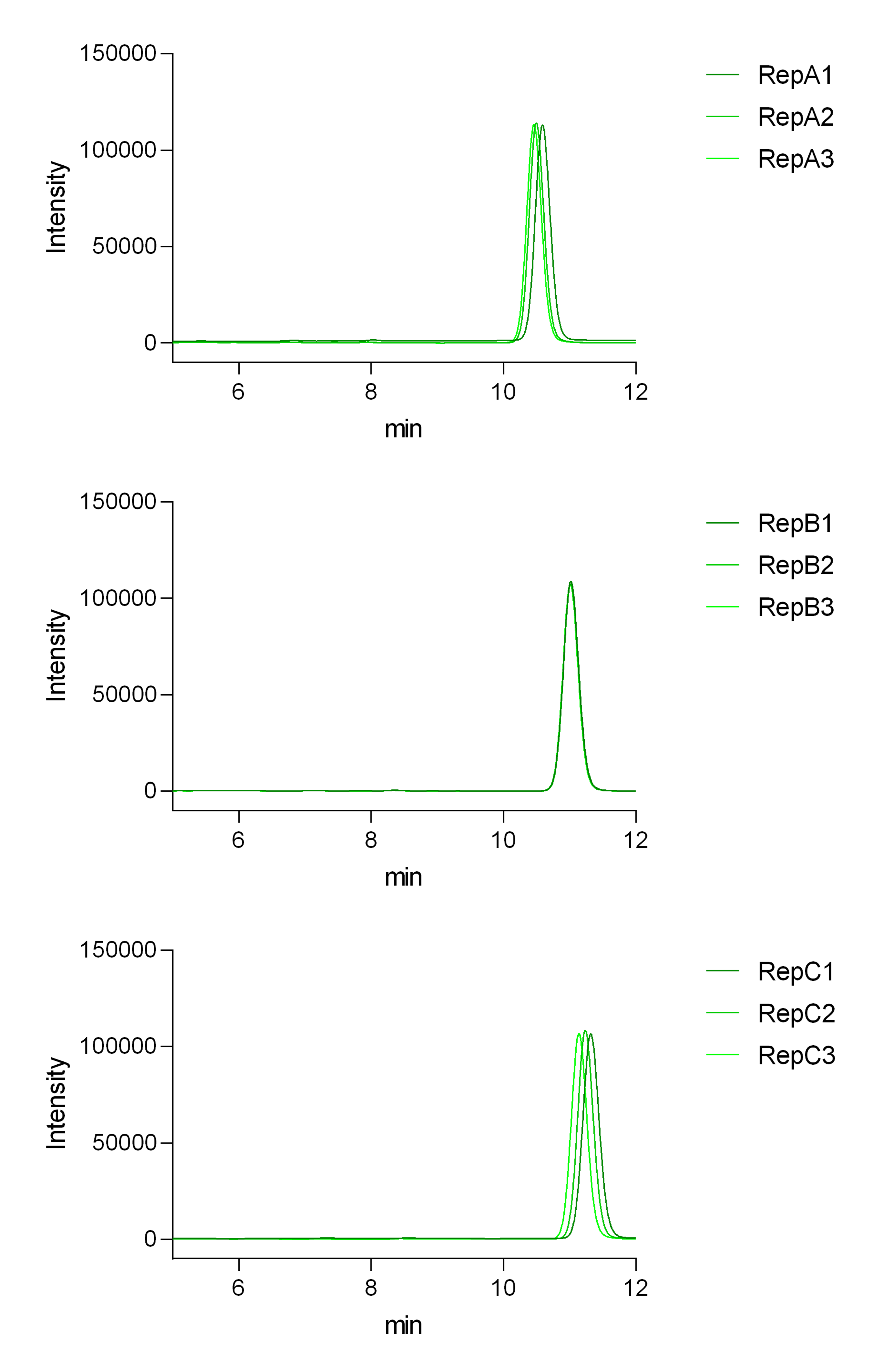

Supplement: Supplementary file 1 [file pharmaceuticals-19-00493-s001.zip › S3 Fig repeatability.tif]

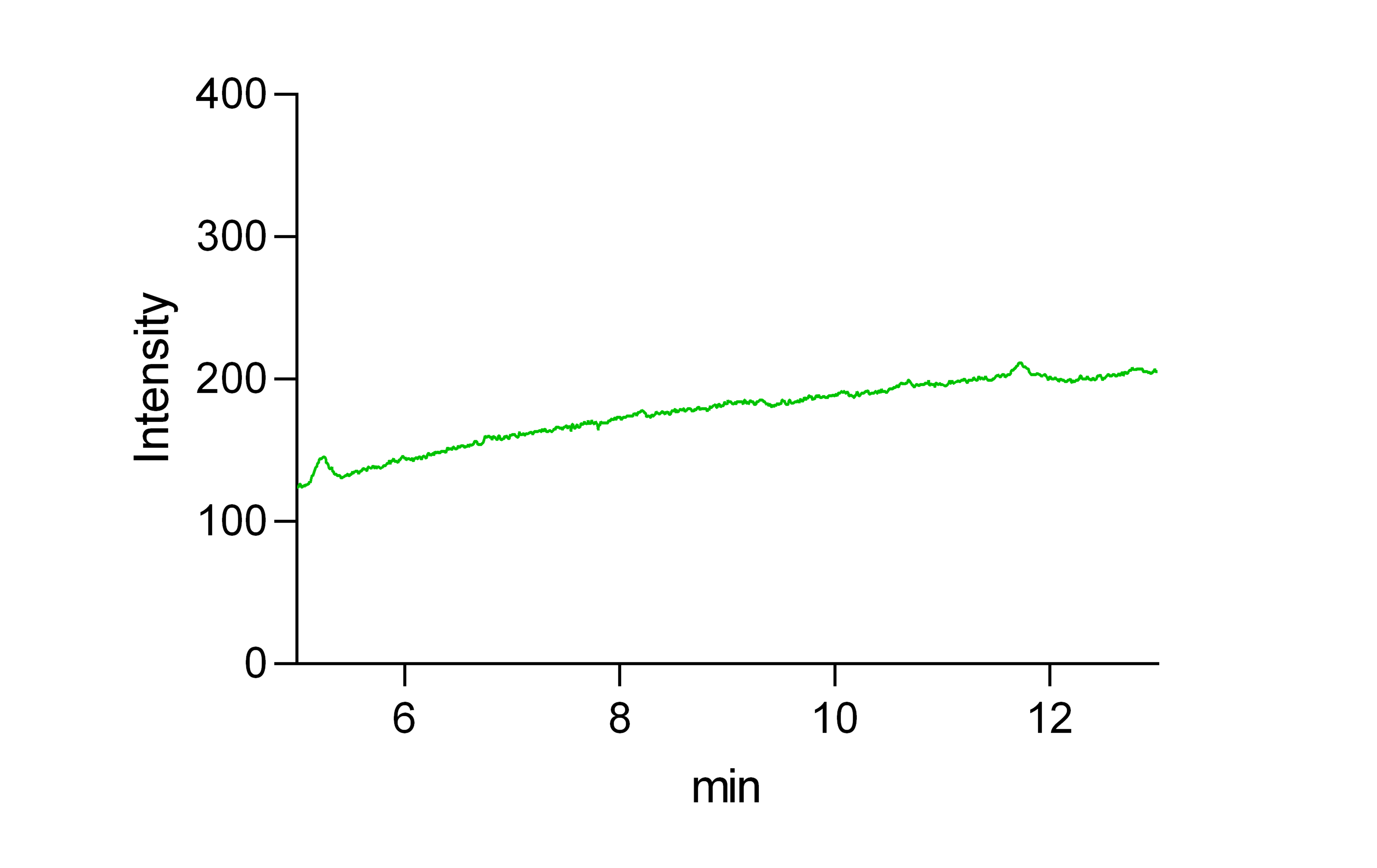

Supplement: Supplementary file 1 [file pharmaceuticals-19-00493-s001.zip › S1 Fig LOD.tif]
